# Recruitment of Transcription Complexes to Enhancers and the Role of Enhancer Transcription

**DOI:** 10.3390/biology1030778

**Published:** 2012-12-05

**Authors:** Jared S. Stees, Fred Varn, Suming Huang, John Strouboulis, Jörg Bungert

**Affiliations:** 1 Department of Biochemistry and Molecular Biology, College of Medicine, Genetics Institute, University of Florida, 1600 SW Archer Road, Gainesville, FL, 32610, USA; E-Mails: jaredstees@ufl.edu (J.S.); fsvarn@gmail.com (F.V.); sumingh@ufl.edu (S.H.); 2 Institute of Molecular Oncology, BSRC Alexander Fleming, Varkiza, 16002, Greece; E-Mail: strouboulis@fleming.gr

**Keywords:** gene regulation, chromatin, transcription factor, noncoding RNA, histone modifications, enhancer, promoter

## Abstract

Enhancer elements regulate the tissue- and developmental-stage-specific expression of genes. Recent estimates suggest that there are more than 50,000 enhancers in mammalian cells. At least a subset of enhancers has been shown to recruit RNA polymerase II transcription complexes and to generate enhancer transcripts. Here, we provide an overview of enhancer function and discuss how transcription of enhancers or enhancer-generated transcripts could contribute to the regulation of gene expression during development and differentiation.

## 1. Introduction

Expression of genes during development and differentiation is regulated by complex mechanisms involving many *cis*-regulatory DNA elements and *trans*-acting proteins [1,2]. Among the *cis*-regulatory DNA elements modulating transcription of genes are promoters, insulators, enhancers and silencers [3,4,5]. This review will focus on enhancer elements that regulate transcription by RNA polymerase II (Pol II) and on the relationship between enhancers and promoters. Promoters are gene proximal regulatory DNA elements that include basal promoter elements [3]. The basal promoter associates with general transcription factors that recruit and position RNA polymerase for faithful transcription. In contrast to promoters, enhancers are usually located far away from the genes they regulate. 

The current estimate for protein coding genes in the mammalian genome ranges from between 24,000 and 25,000 [2]. A recent map of *cis*-regulatory DNA elements in the mouse genome suggests that the number of enhancers far exceeds the number of genes [6]. Many genes are expressed in different cell-types or at different stages during development. Enhancers are often critical for the tissue- and developmental-stage-specific expression of genes and collaborate with promoters to fine-tune the level of expression [5]. Enhancers share many of the hallmarks that characterize promoter regions, including DNAse I hypersensitivity, establishment of a nucleosome depleted region (NDR), binding of tissue-specific and ubiquitous transcription factors, epigenetic marks that signify accessible chromatin and, at least for a subset of enhancers, recruitment of transcription complexes [5]. However, there are differences between promoters and enhancers that we shall discuss later. 

## 2. Enhancer and Locus Control Region (LCR)

Enhancers were first identified based on their ability to increase transcription of a linked gene in transient transfection experiments independent from the orientation of the enhancer with respect to the gene they activate [7,8]. Most viral enhancers, tested in mammalian cells, are ubiquitously active, whereas enhancers of multicellular organisms generally function in a developmental stage-specific and/ or cell-type restricted manner. Like other regulatory DNA elements, enhancers have been traditionally identified by DNase I hypersensitivity mapping experiments [9]. Hypersensitive (HS) sites that were located in relative proximity to Pol II transcribed genes were sub–cloned into reporter constructs and assayed for transcriptional activation using either cell-type specific or ubiquitous promoter elements. These early studies revealed functional differences between different HS sites. For example, some HS sites associated with the human β-globin gene locus control region (LCR) functioned in stable, but not in transient, transfection experiments, suggesting that a regular chromatin environment is important for the function of these elements [10,11]. With respect to overall organization principles, enhancers in many ways resemble promoters. They are about 200 to 400 bp in size and often harbor a number of transcription factor binding sites that recruit either ubiquitously expressed or cell-type-restricted proteins [5]. Enhancers normally do not contain any of the typical Pol II basal promoter elements. like TATA-box, initiator (INI) or downstream promoter element (DPE). However, there are many promoters that also lack these basal promoter elements, yet are capable of recruiting transcription complexes [12].

Locus control regions (LCRs) are composite regulatory DNA elements that regulate multiple genes in complex gene loci [13]. LCRs were first discovered in the human β-globin gene locus and have since been identified in many different gene loci. LCRs differ from simple enhancer elements in that they are usually composed of multiple HS sites that function together to mediate high-level expression of associated genes. LCR associated HS sites can function in an additive, synergistic or in a redundant manner [14,15]. Experiments in the growth hormone gene locus revealed that LCR associated HS sites harbor specific activities that cooperate in transcription activation [16]. Most genes regulated by LCRs are expressed at extremely high levels in differentiated cells [13]. In contrast to enhancers, the LCR associated with the β-globin genes acts in an orientation-dependent manner [17].

**Figure 1:**
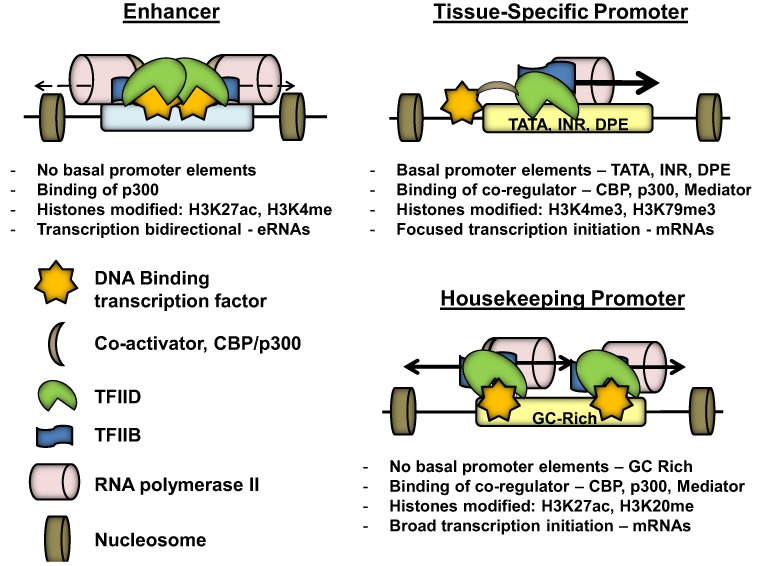
Comparison of the structure and features of enhancer, as well as housekeeping and tissue-specific promoters. Promoters and enhancers are characterized by specific chromatin signatures and recruitment of transcription factors as indicated by the symbols, which are described in the lower right part.

Recent advances in the analysis of chromatin-associated activities using chromatin immunoprecipitation followed by DNA sequencing (ChIP–Seq) or related methods allowed for the genome–wide mapping of transcription factor binding and histone tail post-translational modifications (PTMs) [18,19]. These studies have revealed that enhancer elements can be distinguished from other DNA elements due to a specific combination of histone PTM signatures (Figure 1) [20]. Among these signatures is the monomethylation of lysine 4 of histone H3 (H3K4me), which is enriched at enhancer elements and, to a lesser degree, at promoters. The trimethylated form of H3K4 (H3K4me3) is preferentially associated with promoter regions [21]. The original studies were done with transformed cell lines. Recent studies using primary lymphoid cells also showed enrichment of H3K4me3 at enhancer elements [22]. Importantly however, the ratio of H3K4me1 *versus* H3K3me3 was higher at enhancers compared to promoters. Nucleosomes flanking enhancer elements are often acetylated at H3K27 (H3K27ac) [23,24]. H3K27 is a major substrate for the histone acetyltransferase p300, which is also preferentially found at enhancer elements when compared to promoters [18]. In addition to histone marks, enhancers are occupied by a specific combination of tissue-restricted and ubiquitous transcription factors. Although transcription factors often bind to both promoters and enhancers, some proteins preferentially associate with distal regulatory elements. For example, erythroid transcription factor NF–E2 and the ubiquitously expressed transcription factor USF2 interact with each other and associate with distal enhancer and proximal promoter elements of erythroid specific genes [25,26]. Interestingly, a recent analysis of protein chromatin interaction patterns using a high–resolution DNAse–I based footprinting assay revealed that NF-E2 directly binds to distal elements and recruits USF2 indirectly to these sites; whereas USF2 directly interacts with promoter–proximal sequences and indirectly recruits NF–E2 to these sites [27]. 

At least a subset of enhancers recruit Pol II transcription complexes, including basal transcription factors like TFIIB and TBP [28,29]. Genome-wide RNA sequencing revealed that a large fraction of enhancers are transcribed, often in a bidirectional manner [30,31,32]. The resulting enhancer RNAs (eRNAs) appear to be variable in size and may or may not be polyadenylated. 

There is increasing evidence suggesting that different signatures mark poised enhancers *versus* active enhancers. It has been known for a long time that enhancer elements can exhibit DNase I hypersensitivity in undifferentiated cells in which the target genes are not active [33]. The binding of p300 and the monomethylation of H3K4 of flanking nucleosomes appears to mark poised enhancers, while the recruitment of Pol II, the generation of eRNAs and the acetylation of H3K27 is associated with active enhancers [18,30]. It is likely that there is not a unique combination of signatures that is characteristic for all enhancers. Rather, enhancers likely differ with respect to a specific combination of signatures, which will also reflect differences in activity, e.g. chromatin opening *versus* transcription complex recruitment.

## 3. Promoter

The promoter is a DNA segment that recruits RNA polymerase II with the assistance of promoter binding proteins. The basal promoter is the minimal sequence required for the recruitment of RNA polymerase (Figure 1) [3]. The first isolated mammalian Pol II promoters usually contained a TATA-box located 25 to 30bp upstream of the transcription start site. The TATA-box is recognized by the TATA-binding protein (TBP) [34]. This interaction is stabilized by transcription factor of Pol II (TFII) A and TFIIB [34,35]. TFIIB directly interacts with Pol II and recruits it to the TATA-box protein complex. Additional factors required for Pol II transcription are TFIIE, TFIIF and TFIIH. TFIIH is a large protein complex that contains ATPase and helicase activity required for melting the DNA for transcription initiation. TFII–H also contains kinase activity, which phosphorylates Pol II at the C–terminal domain (CTD) at serine 5 (Pol II-ser-5-P). The genome-wide analysis of promoter structure revealed that only a minority of Pol II transcribed genes contain a TATA-box. Additional basal promoter elements include the initiator (INI) and downstream promoter element (DPE) [36,37]. TBP is part of a larger protein complex called TFIID, which also contains TBP associated factors (TAFs). Some of the TAFs have been shown to interact with the INI and DPE elements, demonstrating that, for many genes, TFIID is the primary DNA binding activity that recruits Pol II to promoters [3]. However, there are many Pol II transcribed genes that do not contain any of the known basal promoter elements, but often are enriched for GC base pairs. Interestingly, promoters with TATA-box or INI elements initiate transcription from very focused sites, whereas GC-rich promoters that do not contain basal promoter elements, like those associated with housekeeping genes, are characterized by dispersed transcription initiation events (Figure 1) [12]. Interestingly, transcription from focused promoters is associated with H3K4me3 and H3K79me1, whereas transcription from GC-rich promoters is associated with H4K20me1 and H3K27ac, a mark also associated with enhancer elements [38].

## 4. Nuclear Transcription Domains

Transcription in eukaryotes takes place in the nucleus. Certain activities in the nucleus, e.g. rRNA synthesis, are compartmentalized. These compartments or domains are not engulfed by membranes, but rather represent dynamic centers of specialized function that are characterized by the constant influx and efflux of activities that constitute these domains. For example, there is evidence that many genes are transcribed in domains enriched for Pol II, referred to as transcription factories [39,40,41]. It appears that transcribed genes associate with transcription factories, while inactive genes do not. Furthermore, in erythroid cells, KLF1 co-regulated genes often associate with the same transcription factory [42]. How transcription domains are established and how genes are recruited to these sites of transcription is currently not known.

The nucleus is also structured with respect to the separation of inaccessible and accessible chromatin. Silent genes are located within inaccessible chromatin territories (CTs), while active genes are located at the surface of these territories [43,44]. Chromatin territories are separated from each other by the interchromatin compartment (IC), viewed as a laguna-like space in the nucleus that contains activities involved in transcription, RNA processing, replication and other functions [43]. The CT-IC model predicts that genes have to be relocated from inaccessible internal domains to accessible domains on the surface of CTs during activation of transcription. What leads to re-positioning of genes away from CTs and anchors them at transcription factories is currently unknown, but enhancers could play a role in this process. 

## 5. Enhancer Function

Enhancers activate gene expression at the level of transcription [5]. Recent evidence suggests that enhancers can be classified with respect to the mechanism by which they increase transcription of target genes [5]. The activities range from mediating chromatin opening, positioning of genes with respect to transcriptionally active regions in the nucleus, recruitment of transcription complexes, to modulating transcription elongation.

**Figure 2:**
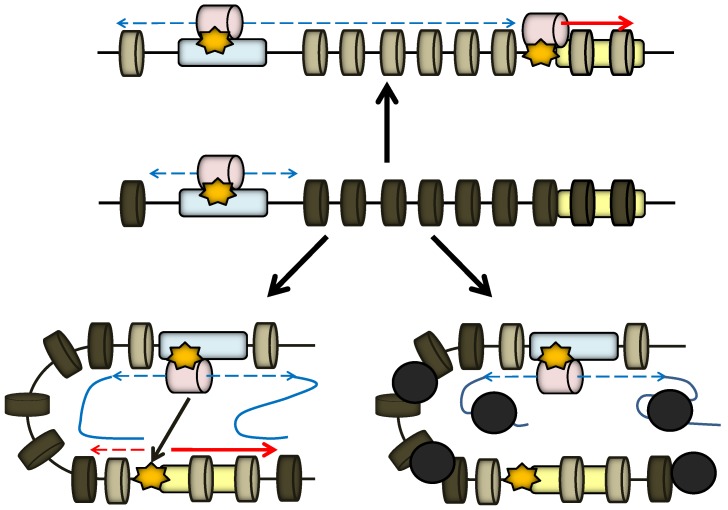
The role of transcription complex recruitment and transcription initiation at enhancers. The diagram in the middle depicts a hypothetical gene locus with a gene (yellow) regulated by an enhancer element (blue). The nucleosomes (small cylinders) are colored dark grey, symbolizing an inaccessible configuration. A transcription complex is recruited to the enhancer, and transcription through the enhancer keeps this element in an open configuration. Upon differentiation, the enhancer activates transcription of the linked gene according to three different scenarios. The mechanism depicted on top shows that tracking of the transcription complex along the chromatin fiber reconfigures the chromatin and renders the promoter accessible, so that transcription factors and RNA Pol II can be recruited to the gene. The model in the lower left indicates that RNA generated from the enhancer and RNA generated from the promoter interact and stabilizes interactions between promoter and enhancer, which facilitates the transfer of activities from the enhancer to the promoter. The model in the lower right indicates that enhancer generated transcripts serve as acceptors for repressor proteins and that the removal of the repressors allows activation of the promoter. The yellow star represents a DNA binding transcription factor. Nucleosomes are represented as light grey (accessible) or dark grey (inaccessible) cylinders. RNA polymerase II transcription complexes are represented as pink cylinders. The blue and yellow boxes represent enhancers and promoters, respectively. The blue and red arrows and lines represent RNAs generated from enhancers (blue) or promoters (red).

It appears that a distinct set of enhancers act by opening chromatin at promoters to increase accessibility for the transcription complex. This has recently been demonstrated for an enhancer element associated with embryonic epsilon globin gene expression in the murine β–globin gene locus [45]. The mechanism likely involves the recruitment of chromatin-modifying activities that spread from the enhancer to mediate accessibility over nearby promoter regions (Figure 2). Another example of enhancer mediated chromatin accessibility stemmed from studies in the α–globin gene locus, which revealed that a distantly located enhancer removes polycomb group (PcGs) complexes from the globin gene promoters [46]. PcG complexes are involved in transcriptional silencing and act by rendering chromatin domains inaccessible [47]. Related to these findings, Taberlay *et al.* [48] demonstrated that the MYOD1 enhancer is in a permissive configuration, marked by a nucleosome-depleted region (NDR) and monomethylation of H3K4me1 in undifferentiated cells, while the gene it regulates is associated with PcG complexes and H3K27me3, a modification introduced by the histone methyltransferase Ezh2, a component of PcGs [47]. During differentiation into muscle cells, master regulators (like Myod1 and Oct4) bind the enhancer and recruit activities that ultimately remove H3K27me3 and PcG from promoters. 

Enhancers have been shown to directly recruit transcription complexes, which could be delivered to genes by looping mechanisms [29,49,50,51]. A variation of this theme is that enhancers may either recruit specific gene loci to preforme transcription factories in the nucleus or actually initiate the formation of these domains. It was shown that the β–globin genes do not associate with transcription factories in erythroid cells in the absence of the LCR [52]. Other studies have shown that enhancers are important for the relative position of genes in the nucleus. Again, the β-globin LCR was shown to be required for the re-positioning of genes toward the surface of chromatin territories upon activation [53].

There is also increasing evidence demonstrating that enhancer elements affect elongation of RNA Pol II by mediating the recruitment of transcription elongation factors like pTEFb or components of the FACT (facilitator of chromatin transcription) complex [54,55,56]. This could be related to the ability of enhancers to recruit genes into transcription factories, which may be enriched for these activities. Enhancer mediated activation of transcription elongation could play an important role in the activation of gene expression during differentiation of embryonic stem cells [56]. Many developmental stage–specific genes are repressed in ES cells, but nevertheless were shown to be associated with Pol II transcription complexes [57]. Upon differentiation along specific cell lineages, enhancer elements could switch on paused Pol II transcription complexes at developmental gene promoters. 

The above described examples illustrate that enhancers are quite diverse with respect to their roles in gene activation. For genes regulated by multiple enhancers, each enhancer could provide a unique activity, and the collective set of enhancers could therefore mediate optimal gene expression. 

## 6. Transcription Initiation at Enhancers

The hitherto clear distinction between promoters and enhancers has become blurry due to recent evidence showing that enhancers are capable of recruiting transcription complexes and a recent demonstration that promoters can activate other promoters [58,59]. Tuan and colleagues demonstrated in 1992 that HS2 in the human β-globin LCR recruits Pol II transcription complexes, and the authors detected long non-coding transcripts downstream of HS2 [60]. These results suggested the possibility that Pol II is first recruited to long distance regulatory elements and tracks along the chromatin until it is positioned at promoters to generate protein coding mRNAs. A tracking mechanism was supported by later studies demonstrating that an insulator placed between the LCR and the genes reduced expression of the globin genes and led to accumulation of Pol II at the insulator element [61]. In addition to HS2, other β-globin LCR associated HS sites also recruit Pol II and harbor promoter activity [62,63]. Studies by the Paro laboratory showed that transcription through a polycomb group responsive element (PRE) in *Drosophila* switched the element from being silent to being activated [64]. The continuous transcription of the PRE element prevented PcG mediated silencing. One possible explanation of these results is that the process of transcription erased specific signatures at the PRE elements and allowed establishment of a different set of signatures. Transcription mediated modification of chromatin structure by Pol II-associated co-regulatory protein complexes has been demonstrated for other gene loci as well [65,66].

Intronic enhancers have also been shown to recruit transcription complexes, which led investigators to propose the term "prohancer" for those enhancer elements that also exhibit promoter activity [67]. A recent study by the Higgs laboratory demonstrated that, in fact, many intronic enhancers have promoter activity and confer alternative transcription start sites for protein coding genes [68]. 

Despite earlier findings of enhancer mediated Pol II recruitment, it was a surprise when in 2010 the Greenberg laboratory showed that thousands of neuronal specificity enhancers recruited Pol II and initiated formation of enhancer RNAs (eRNAs) [30]. The eRNAs were relatively short and resulted from bidirectional transcription initiated in the enhancer elements. Other laboratories have confirmed these studies, demonstrating that enhancer mediated recruitment of Pol II is a common event [31,32]. A recent investigation into the global nature of eRNAs revealed that most of these RNAs remain in the nucleus [69]. Furthermore, comparing the results of global high throughput RNA-sequencing (RNA-Seq) and of Hi-C, a technique that assays proximity between regulatory elements genome-wide, demonstrated that the occurrence of eRNAs correlated with the proximity between enhancers and promoters, suggesting that these transcripts are associated with active enhancers [70].

## 7. Possible Functions of eRNAs

Non-coding RNAs have been shown to associate with protein complexes and to directly regulate expression of genes [71]. For example, it was shown that non-coding RNAs associate with PcG complexes and silence expression of specific target genes [71]. In this regard, enhancer-associated non-coding RNA could serve to tether PcG complexes away from promoters, as outlined in Figure 2. Other studies have shown that non-coding RNAs transcribed from HS sites associate with co-activators and stimulate transcription of genes [72,73]. Curiously, the non–coding RNAs even functioned in transient transfection assays on heterologous promoters despite the fact that *in vivo* they appeared to regulate specific target genes [72]. However, this is also a characteristic of some enhancers.

There are many studies showing that RNAs are involved in the formation of specific protein/ RNA complexes or nuclear domains [74]. The ribosome is one example, in which extensive RNA/ RNA and RNA/ protein interactions provide the frame-work for protein synthesis. In addition, Shertsov and Dundr demonstrated that RNAs are involved in nucleating the formation of nuclear histone bodies in the nucleus, which are sites of histone gene transcription [75]. Therefore, the role of many non-coding or eRNAs could be to nucleate or stabilize specific nuclear structures, like transcription, replication or repair domains. In this respect, it is interesting to note that histones associated with double strand breaks are associated with methylated H3K36, a signature of transcribed chromatin [76]. Taking this idea a step further, RNA could also be involved in promoting long-range interactions between enhancers and promoters (Figure 2). Conventional or non-conventional base-pairing between RNA molecules tethered to enhancers and promoters could mediate or stabilize promoter/ enhancer contacts. RNA base-pairing-mediated interactions between enhancers and promoters would not only provide stability, but also specificity.

## 8. Poised Enhancers During Cellular Differentiation

Different classes of enhancers can be distinguished by their specific epigenetic signatures. Zentner *et al*. [24] reported that active enhancers are either marked by H3K4me1/ H3K27ac or by H3K4me1 and lack acetylation of H3K27. Genes that are associated with enhancer containing the H3K27ac mark are expressed at higher levels compared to those associated with enhancers that only harbor the H3K4me1 mark. The authors thus distinguish active from intermediate enhancers based on these different modifications and differences in expression levels of associated genes. Poised enhancers are also associated with H3K4me1, but lack H3K27ac. Many poised enhancers are associated with H3K27me3 or H3K9me3, as well as with unstable nucleosomes containing the histone variants H2A.Z and H3.3 [24,59]. Interestingly, the active enhancers are characterized by the presence of Pol II, phosphorylated at serine 2 and 5 of the CTD, as well as H3K36me3, indicating active transcription at these elements [24].

Enhancers are often in a poised configuration in progenitor or stem cells [77]. In fact, many enhancers regulating early developmental transcription factor genes are associated with bivalent chromatin-containing histone modifications typically associated with active (H3K4me3) or silent (H3K27me3) genes in embryonic stem cells [77]. Differentiation of these cells into specific lineages leads to the erasure of either the H3K4me3 or the H3K27me3 mark, depending on whether an enhancer is active or inactive, respectively. Other enhancers are activated later during differentiation and usually associate with so-called pioneer-transcription factors that initiate chromatin remodeling at these elements [78]. In general, it appears that the reconfiguration of chromatin structure at enhancers precedes the activation of promoters. This is supported by recent findings demonstrating that nucleosomes flanking poised enhancers are marked by H3k9me3 [79]. These marks could be bound by transcription factors that subsequently activate enhancers [27]. 

Observations that enhancers are often primed in progenitor or stem cells suggest that these elements are the primary site of recruitment for activities involved in transcription activation, be it activities that open chromatin, activities that recruit transcription complexes to target genes or activities that stimulate transcription elongation. Moreover, a recent report demonstrated that an enhancer associated with the T-cell-specific CD4 gene is required for initiating activation of the gene locus in undifferentiated cells that do not express the target gene, but is no longer required in differentiated cells that express the CD4 gene [80]. Likewise, DuBose *et al.* [81] demonstrated that deletion of the SNRPN imprinted control region during early embryonic development causes imprinting defects of the associated genes; however, deletion of this element in adult brain cells has no effect on imprinted gene expression. These studies support a previous finding from the Schaffner laboratory showing that the B-cell-specific IgH enhancer is only transiently required for setting up the active state of the target gene [82]. Based on these findings, it is possible that enhancers initiate the formation of an active gene locus and that epigenetic signatures that are introduced during activation are propagated through subsequent differentiation steps. The process of transcription initiated at enhancers could contribute to establishing an activated state that is propagated through subsequent cell divisions. 

**Figure 3:**
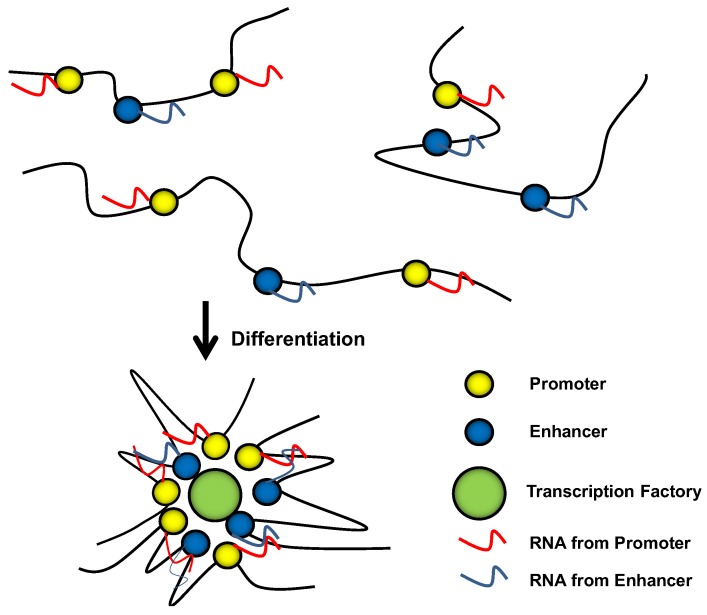
Enhancers, promoters and transcription factories. It is proposed that upon activation of genes during cellular differentiation, enhancers and promoters come together to establish transcription factories. Cell-type specific transcription factors bound at promoters and enhancers are involved in mediating proximity between the elements and in the recruitment of transcription complexes. RNAs generated from enhancers (blue) and promoters (red) stabilize formation of transcription domains.

As mentioned before, recent interaction maps reveal extensive enhancer/ promoter and promoter/ promoter interactions [58]. RNAs generated at promoter and enhancer regions could be involved in regulating associations between the regulatory elements (Figure 3). It is possible that enhancers and eRNAs are involved in setting up transcription domains that are propagated through subsequent cell divisions by epigenetic signatures, so that in differentiated cells, promoter/ promoter interactions could substitute for enhancer/ promoter interactions [58].

## 9. The Role of Enhancer-Mediated Transcription in Chromatin Modification

It should be pointed out that the overall level of sequence conservation of non-coding RNA is 10-fold lower compared to mRNA [31]. Furthermore, it was found that upstream extragenic transcription precedes activation of gene transcription, that extragenic transcripts are generated at a level 100-fold lower than average gene transcripts and that these extragenic transcripts are unstable [31]. Therefore, aside from generating eRNAs, the process of enhancer initiated transcription in itself could aide in generating or maintaining an open chromatin configuration during cellular differentiation (Figure 2). This could either involve large chromosomal domains or could be restricted to the vicinity of enhancers. Studies in the human growth hormone gene locus demonstrated that noncoding transcription, rather than noncoding transcripts, mediate long-range enhancer function [83]. Furthermore, enhancer transcription could be involved in introducing the H3K27ac mark, as both of these events mark active enhancers. Another possibility is that low level transcription of enhancers in undifferentiated cells could maintain a dynamic semi-open state that could be stabilized by increased expression of cell-type specific transcription factors in differentiated cells. In this respect, it is important to point out that transcription of promoters with dispersed initiation sites often occurs in both directions similar to what has been described for enhancer transcription [84]. This divergent transcription could aid in shifting nucleosomes away from these regulatory regions. Transcription of enhancers could thus facilitate the formation of cell-type specific enhanceosomes [85].

## 10. Conclusions

The recent genome-wide analysis of histone PTMs and transcription factor binding revealed novel aspects of long-distance regulatory elements. Enhancers and gene proximal promoters share the ability to recruit transcription complexes and to regulate expression of genes over long distances. In hindsight, this may not be too surprising, as enhancers and promoters often harbor similar arrangements of transcription factor binding sites. However, in contrast to transcription at enhancers, it appears that promoter-mediated transcription is more efficient and leads to the production of stable RNAs. The reasons for this are currently not known, but may be due to the fact that promoters contain basal promoter elements that recruit Pol II transcription complexes with a high affinity. Furthermore, promoters generate transcripts that are conserved and contain sequences that mediate stability. It will be important to determine if transcription complex assembly at enhancers follows similar pathways as those operating at promoters. Other outstanding questions pertain to the role eRNAs may play during activation of transcription and if the process of transcription also contributes to the function of enhancers. The available data so far suggest that enhancers exhibit different activities. Therefore, it is possible that for those enhancers that are involved in modulating chromatin domains, the process of transcription is important, while for those enhancers that modulate the recruitment or activity of transcription complexes, the eRNA transcripts directly participate in the activation process. 
